# Synergistic Effect of Polycarboxylate Superplasticiser and Protein Retarders in Cementitious Materials Containing Na- Montmorillonite: Effect of Addition Methods

**DOI:** 10.3390/ma15196614

**Published:** 2022-09-23

**Authors:** Zhenhe Tian, Jun Ren, Hao Li, Xusheng Wang, Yang Feng, Wei Xiong, Jialing Yang, Shengye Xu, Zengle Ren

**Affiliations:** 1 China Construction Seventh Engineering Division Corp., Ltd., Zhengzhou 450000, China; 2 Urban Construction and Digital City Teaching Experiment Center, School of Architecture and Planning, Yunnan University, Kunming 650550, China; 3 Guangdong Power Grid Energy Development Co., Ltd., Guangzhou 510000, China; 4 Guangdong Provincial Key Lab of Robotics and Intelligent System, Shenzhen Institute of Advanced Technology, Chinese Academy of Sciences, Shenzhen 518055, China

**Keywords:** polycarboxylate superplasticiser, protein retarders, montmorillonite, addition method, compatibility

## Abstract

Polycarboxylate superplasticiser (PCE) is notably sensitive towards Na-Montmorillonite (Na-Mmt), an impurity generated from the manufacturing of concrete aggregate due to the chemical intercalation and poor surface adsorption. In order to improve the poor compatibility of PCE, the protein-based retarders were applied as the sacrificial agents, and its synergetic effects in cementitious materials containing Na-Mmt were investigated. The protein-based retarders were applied as the sacrificial agents and its synergetic effects in cementitious materials containing Na-Mmt were investigated. In addition to test rheology, minislump, and setting time, the adsorption behaviour and intercalation were characterised via Total Organic Carbon, X-ray photoelectron spectroscopy, and X-ray diffraction. The results revealed that the incorporation of protein-retarders improved the performance of PCE in terms of workability, and the rheological behaviour of cement with Na-Mmt. Moreover, compared to simultaneous addition, the application of separate addition further increased the workability and improved workability retention, with best dispersion performance obtained by prior adding the retarders, which could be due to the lessened intercalation between the layers of Na-Mmt.

## 1. Introduction

The addition of chemical admixture is an effective method to improve the performance of Portland cement (PC)-based concrete. Polycarboxylate superplasticizer (PCE) has been widely used due to its excellent performance in reducing water consumption of concrete and improving the workability and fluidity of concrete [1,2]. Generally, the PCE is a type of comb shape or brush-like structured water-soluble polymer, composed of a backbone with different anchored groups for adsorbing on particle surface and the polyethylene glycols (PEO) based side chains, including α-allyl-ω-hydroxy poly (ethylene glycol) ether (APEG), 2-methyl allyl polyoxyethylene ether (HPEG), isoprenyl oxy polyethylene glycol ether (IPEG), and methoxy polyethylene glycol methacrylate (MPEG), etc., which can physically hinder the agglomeration from cement particles [3,4,5]. It has been widely accepted that the performance of PCE in concrete is usually achived by three apporaches in terms of the adsorption of polymers on particle surface: the dissolution of polymers in aqueous phase and the intercalation or coprecipitation within the raw material or hydration product [6,7,8]. It should be noted that among the different approaches, the adsorption, in which the polymer was adsorbed on the surfact according to electrostatic and/or chemical coordination attraction, is important for the dispersion performance of PCE [9].

Recently, due to the rapid development of infrastructure construction, aggregates contaminated by impurities, such as silt, clay, and stone powder, have been inevitably used [10,11]. While PCEs have showed a better performance in PC-based materials compared to other type of superplasticisers, it has been widely accepted that the PCEs are less effective in the presence of clays, such as montmorillonite, kaolinite, and Muscovite [12,13], which has been confirmed by the multi-fold dosage requirement of PCEs to achieve the same mobility [14].

According to the currently research on the mechanism of incompatibility of PCE with clay, the significantly reduced dispersion effect of PCEs with clay has been reported [15,16,17]. The main reason is the larger surface area of clay, which not only adsorb mixing water but also leads to the increased adsorption of PCE molecules [18]. Moreover, the two silicon tetrahedrons and one aluminium oxide octahedron composed structure of the clay can lead to the expansion of the interlayer space after it contacts the water [19,20], which may trap the PEO side chains of PCEs and defunctionalize the steric repulsion of PCEs in the dispersing cement particles [21].

Among different type of clays, the montmorillonite (Mmt) has been identified as the main gradient to suppress the improvement on fluidity of concrete by PCEs [22,23]. It has been reported that Mmt has the chemical formula of Mx (Mg, Al, Fe)_2_ (OH)_2_ [Si_4_O_10_]·nH_2_O, with a 2:1 type phyllosilicate mineral formed alternately by alumina octahedra and silica tetrahedra [24]. Therefore, due to its unique crystal structure, Mmt exhibits different physicochemical properties such as cation exchange properties, negatively charged layer, layer electronegativity, and water swelling, which affects the hydration of cement paste. In addition, compared with PC, Mmt exhibits a higher specific surface area which greatly impacts the interaction of PCEs and the workability of cement [25].

Generally, the interaction of PCE with Mmt can be divided into the adsorption on surface and intercalation from interlayers, the latter of which showed no contribution to the dispersion of PCEs [26]. As in the cementitious system, the adsorption of negatively charged PCEs to positively charged clay surfaces by coordination with Ca^2+^ ions to form the adsorption layer is one of the functions contributing to the dispersion [27,28]. However, different from that in PC, the intercalation has been identified as a main sorption behaviour of PCE when it is mixed with Mmt due to their sandwiched structured between the inter-layers, which allowed the oxygen atoms in PCE to produce hydrogen bond complexation with the water molecules in those space [29,30]. It has been reported that 70% of PCEs were identified to act with Mmt by intercalation [31]. Therefore, this intercalation led to a ~100 times higher sorption of PCEs on Mmt other than cement grain [32].

In order to solve the problem of incompatibility between PCE and Mmt, many methods have been proposed. These methods involve adjusting the structure of PCE polymers, i.e., changing the length of PCE side chains, replacement of the new side chains, etc. [33,34,35], and combining with other admixtures, for example, sacrificial agents, has been attempted [36,37]. Compared to the development of novel clay-tolerance PCEs, the addition of sacrificial agents, a compound mitigating the interaction between PCEs and clays, in terms of intercalation blockers, clay swelling inhibitors, electrostatic blockers, or combined functionalised agent, has been widely applied due to its cost and ease to be achieved [38]. Therefore, different chemicals, i.e., polyether, polymeric ferric sulphate, sodium tripolyphosphate, etc., have been borrowed to be the sacrificial agents [22,26,39,40]. Tan et al. [22] improved the clay tolerance by incorporating polymeric ferric sulphate, which was reported to block intercalation of PCE, but also significantly increased surficial adsorption. In another study [40], sodium tripolyphosphate was used as a sacrificial agent to increase the dispersibility of PCE, and it was found that with the increase of sodium tripolyphosphate content, the dominant factor changed from dispersion to intercalation. Furthermore He et al. [41] and Yang et al. [42] used different sacrificial agents, and the dosage was considered. With the change of dosage, tolerance was improved in varying degrees. Although it has been confirmed that the type and dose of sacrificial agents will have a greater impact on the effect, other factors have rarely been studied.

As another important category of admixture, the retarders has been used to regulate the setting behaviours in a cementitious system with fast hydration [43,44,45]. To achieve multiple performances in adjusting the properties of cement, the retarders are often combined with the PCEs in a Portland cement-based system or those novel cementitious systems [46,47]. However, in cementitious system with clays, the combination of PCE with retards has not been extensively research. Moreover, it should be noted that, in almost all the cases, the PCEs are added simultaneously with the retarders. Since it has been proved that the change of addition sequence of admixture can benefit and further improve the performance of PCEs [48,49,50], by borrowing this concept, the application of different addition methods on retarders and PCEs could potentially improve the dispersion performance of PCE in the cement system with Mmt.

Therefore, the purpose of this study is to explore the effect of addition methods of water-reducing type PCEs and protein-based retards on the dispersion of cement system with different substitution rate of Na-montmorillonite (Na-Mmt). The influence of different addition methods, in terms of simultaneous addition, delayed addition and prior addition, on the minislump and rheology of cementitious paste was observed. Through XRD and adsorption effect test, the adsorption and intercalation behaviour were observed, and its mechanism was discussed. This study provides a novel method for the use of sacrificial agents and promotes the development of sacrificial agents in engineering applications.

## 2. Materials and Methods

### 2.1. Materials

#### 2.1.1. Cement and Montmorillonite

The P.O. 42.5 cement used in this experiment is provided by China United Cement Co. Ltd. Sodium-based MMT (Na-Mmt) is provided by Shenzhen Huanan Xinyang Tech. The typical chemical compositions of the cement and clay are listed in Table 1.

#### 2.1.2. PCE and Retarders

**Figure 1:**
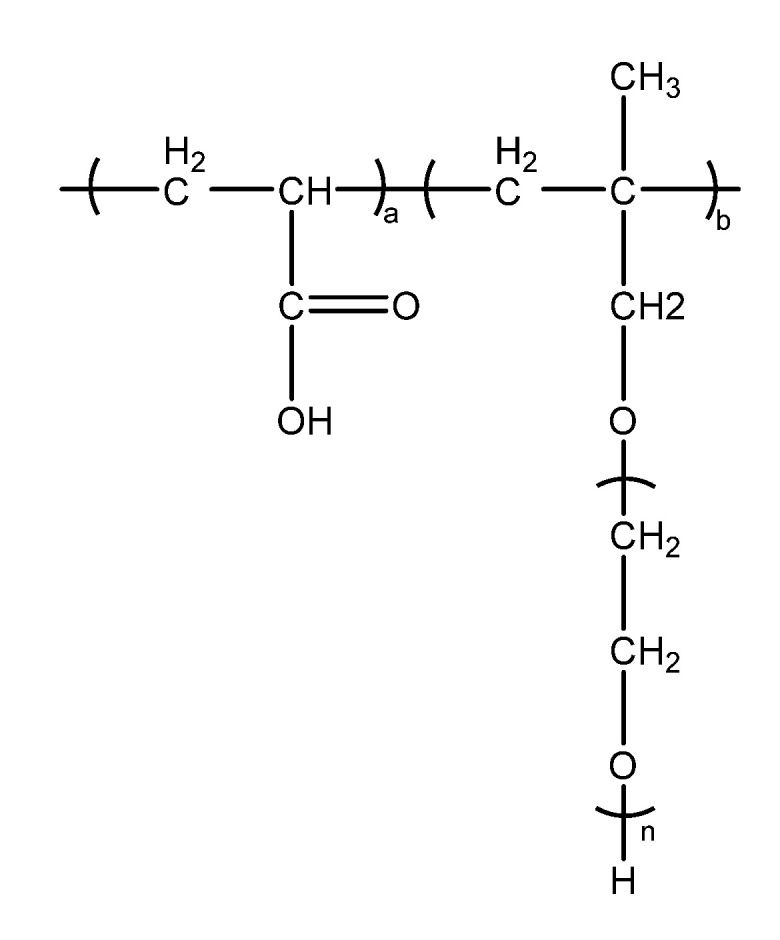
Chemical structures of the PCE.

The market-available water-reducing type of polycarboxylate superplasticiser (mother liquors with solid content of 40%) was used in this paper, supplied by KZJ New Materials Group Co., Ltd. The schematic of chemical structure of the PCE, provided by the manufacturer, is shown in Figure 1, in which the PCE is composed of methyl allyl polyoxyethylene ether (HPEG-2400) and acrylic (AA). The protein-based alkaline retarders 7602 (PR for short hereafter) were provided by Bonuo New Materials (Beijing) Technology Co., Ltd.

### 2.2. Sample Preparation

The details of mixes are presented in in Table 2. The cement paste was prepared in accordance with Chinese standard GB/T 8077–2012, in which the water to cement (w/c) ratio was fixed at 0.29. The substitution rate of Na-Mmt was set as 0%, 3%, 5%, 7% by weight of the cement. The dosage of PCE (added based on the solid phase of PCE) and PR was respectively controlled at 0.6% and 0.05% (by the mass of total cementitious materials). Three different approaches of adding PCE and PR were investigated, which are named accordingly as: (1) simultaneous addition (SA): adding PCE and PR together when mixing with PC; (2) delayed addition (DA): adding PR with 2/3 mixing water to cement first and then PCE with 1/3 mixing water at a 3 min interval; and (3) prior addition (PA): adding PCE with 2/3 mixing water to cement first, then PR with 1/3 mixing water at a 3 min interval. The 3 min interval was selected based on the preliminary trials and based on a minislump test, in which the 3 min showed the best performance.

### 2.3. Minislump

The minislump of the cement paste was conducted by a mini cone according to Chinese standard GB/T 8077–2012, in which the average of two diameters under perpendicular direction was reported. The minislump was conducted at 5, 30, and 60 min after mixing.

### 2.4. Rheology Test

The rheological behaviour of the PCE incorporated cement pastes was determined at 5, 30, and 60 min after the mixing to obtain the shear stress and the shear rate curve by Lamy Viscometer RM100, which was followed by the previous study [51]. During the test, the shear rate was increased from 10 to 150 s^−1^ for 40s, and the measurements were conducted at 10, 30, 50, 70, 90, 110, 130, and 150 s^−1^ for drawing the up-curve test and at 130, 110, 90, 70, 50, 30, and 10 s^−1^ for the record of down-curve. Before testing, two repeated tests of the cement were conducted to determine the repeatability of the measurement. The thixotropy area of the cement was calculated using Chen’s method [43].

### 2.5. Setting Time

The setting time of the specimen was examined according to the Chinese standard method GB/T 1346-2011.

### 2.6. Adsorption Test

The adsorption amount of PCE with Na-Mmt was determined by Total Organic Carbon (TOC) Equipment (Multi N/C 2100 Jena, Germany) followed by the previous study [52]. For testing, a mixture of 0.50 g Na-Mmt, 24.50 g of synthetic cement pore solution (prepared by dissolving 1.72 g CaSO_4_·2H_2_O, 4.76 g K_2_SO_4_, 6.96 g Na_2_SO_4_, and 7.12 g KOH in 1 L of deionized water [53]) and the according amount of PCE were added into a 50 mL container and stirred for 9 min. The mixture was then centrifuged to separate the supernatant for a TOC test. The adsorption amount differences before and after contact with Na-Mmt was the adsorption amount of PCE.

The adsorption layer thickness of PCEs on Na-Mmt surface was calculated from Si2P analysis from X-ray photoelectron spectroscopy by following the previous method [40,51]. The prepared solid powders were vacuum dried before X-ray photoelectron spectroscopy (Thermo Scientific K-Alpha, Thermo Fisher Ltd., Waltham, MA, USA) measurements. The silicon was tested with the aluminium as an anode target under the energy resolution of 0.05 eV.

### 2.7. XRD Test

The interlayer spacing (d-spacing) of multiple layers of Na-Mmt with PCE were determined by XRD with CuKα radiation (D8 type XRD machine by Bruker, Germany) [52]. The characterisation was conducted with the diffraction angle (2θ) between 2° to 40° under a scanning speed of 2°/min.

## 3. Results and Discussion

### 3.1. Effect on Minislump

**Figure 2:**
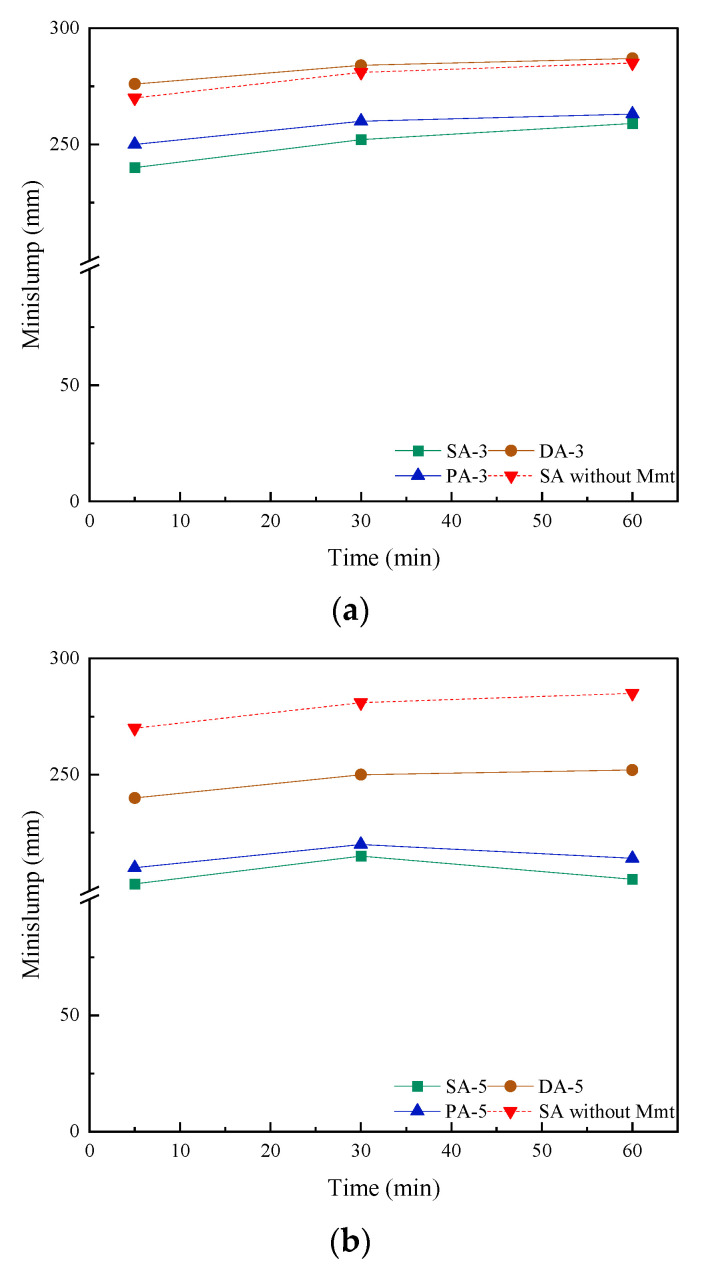

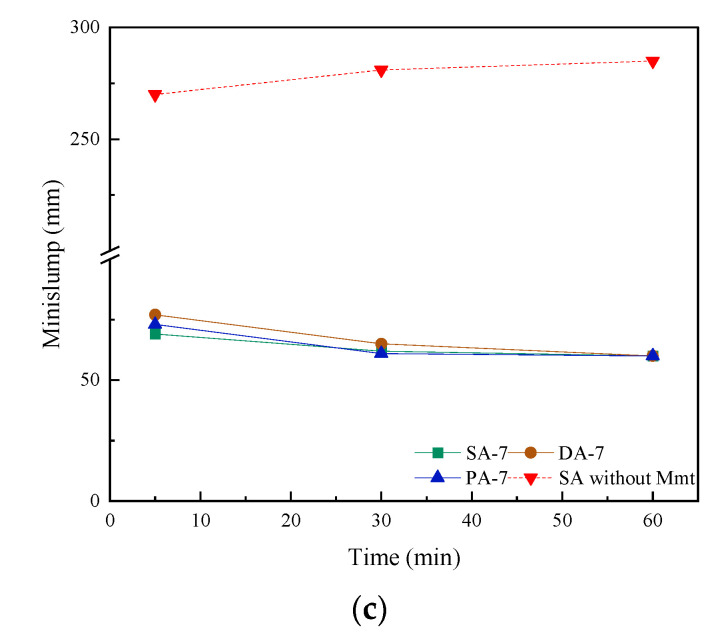
Effect of different addition methods on the minislump of cement paste with different substitutions of Mmt: (**a**) 3% Mmt substitution rate; (**b**) 5% Mmt substitution rate; (**c**) 7% Mmt substitution rate.

The effect of different adding methods of PCE and PR on the workability of Na-Mmt substituted cement paste was measured by a minislump test, and the results are shown in Figure 2. It can be seen from the figure that the substitution of the Na-Mmt significantly reduced the minislump of the cement paste incorporated with PCE, for example, compared to the PCE incorporated cement without any Na-Mmt, the initial minislump was 271 mm. However, it decreased to 235 mm, 210 mm, and 62 mm when substitution rates were 3%, 5%, and 7%, respectively, which indicated that the addition of Na-Mmt could significantly affect the performance of PCEs. This is consistent with existing research results [54,55]. Without PCE, the addition of montmorillonite has led to a decrease in workability. After adding PCE, the difference in workability was more obvious due to the tolerance of Mmt. This is also the reason why there is such a big gap with the cement without replacing Mmt when the content reaches 7%. However, when the separate addition methods, in terms of prior addition (PA) and delayed addition (DA), were applied, the performance of the PCE improved. This phenomenon was more obvious when the substitution rate was low (below 5%). For example, when the substitution rate was 3%, the application by PA and DA increased the initial minislump from 240 mm (by SA) to 250 mm and 276 mm, respectively, which indicated that the workability of the cement paste with Mmt was improved. It should be noted that under DA, the initial minislump was even slightly higher than that of cement paste without Mmt (270 mm), revealing that the negative effect from Mmt was suppressed. However, it should be noted that, in the unusual case of the substitution rate being higher than 7, although the increment by separate addition could be observed, the improvement was not obvious.

Moreover, regarding the slump retention, the separate additions of PCE and PR can improve the workability retention as well. For example, the minislump at 5 and 60 min of 5% Mmt replaced cement paste under SA were 203 mm and 205 mm, respectively. However, when a separate addition was applied, in particularly by the DA, those values changed to 240 mm and 252 mm, in which the delayed plasticising effect was observed, indicating an improved performance of PCEs in cementitious materials [56]. Similar to the initial minislump, although the separate addition could improve the performance of PCEs with Mmt, the improvement by the separate addition on workability retention was still confined when the Mmt substitution rate was higher than 7%, which does not often happen in practice.

### 3.2. Effect on Rheology Behaviour

**Figure 3:**
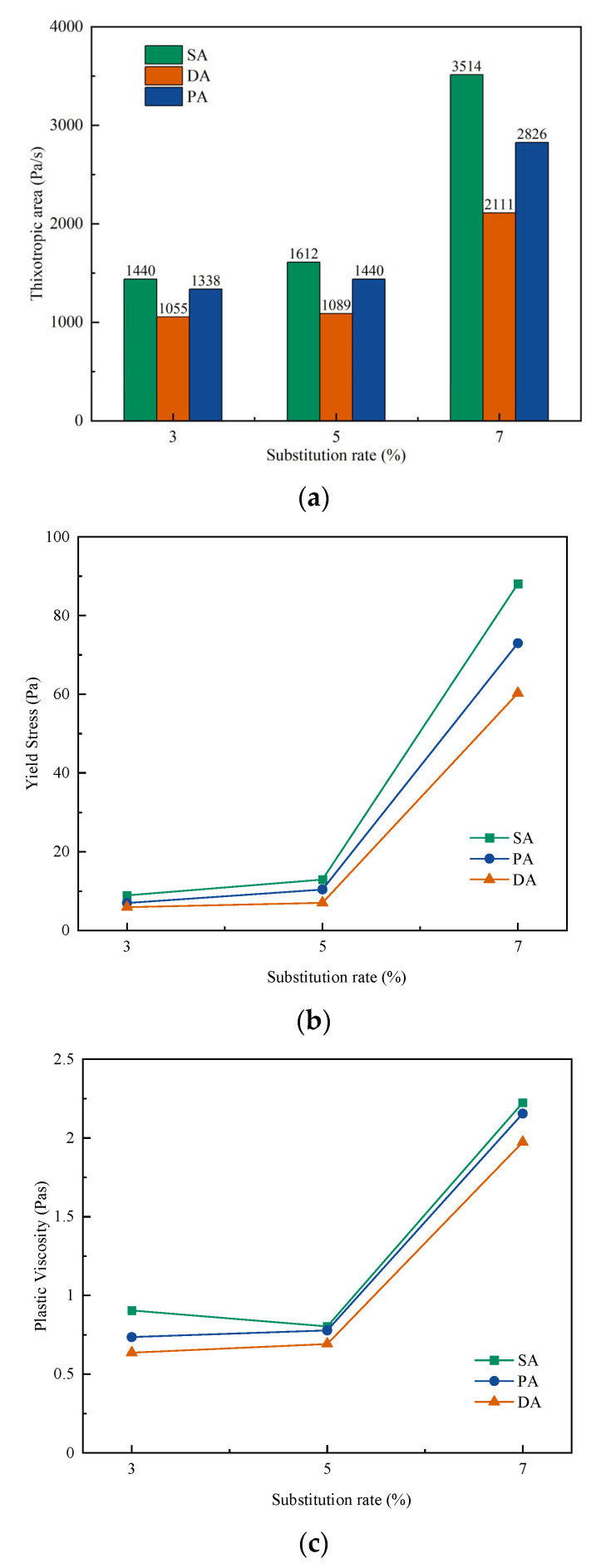
Rheological behaviour of cement pastes with PCE and PR under the different substitution rate of Na-Mmt: (**a**) thixotropic area; (**b**) yield stress; (**c**) plastic viscosity.

The rheological parameters generated from the flow curves, in terms of thixotropic area, yield stress, and plastic viscosity, of the three admixtures addition methods with different substitution rates of cement with Na-Mmt are shown in Figure 3. It can be seen from Figure 3a that by increasing the substitution rate of Mmt, the thixotropic area of the cement paste with PCE and PR was increased. In particular, when the substitution went from 5% to 7% under SA, the area increased from 1613 Pa·s to 3536 Pa·s. However, as with all substitution rates, when the separate addition method was applied the thixotropic area was decreased, with lower values obtained from the DA method. Because the thixotropy behaviours relate to the structural build-up and the structural breakdown of the paste under shearing, the reduced results indicated that the DA method of PCE and PR could lead to good dispersion of cement particles and may hinder the intercalation process with Mmt [57,58], which will be discussed later.

Moreover, similar to the effect on thixotropic area, as shown in both Figure 3a,b, the increase of Mmt substitution rate led to an increase of both yield stress and plastic viscosity of the cement paste, excepting the plastic viscosity by SA, in which the plastic viscosity slight reduced with an increasing substitution rate from 3% to 5%. This experimental result is similar to the conclusions from Ma et al., [59], in which the effect of a variety of different PCEs without adding sacrificial agents was investigated, and results that the yield stress and plastic viscosity increased with the increase of the substitution rate, increased slowly between 1% and 2%, and increased rapidly between 2% and 3%, were reported. It can be seen that the addition of a sacrificial agent did not change the trend that the yield stress and plastic viscosity increased slowly and then increased rapidly with the increase of substitution rate. Similar to the effect on workability, the application of separate addition also decreased both yield stress and plastic viscosity of the cement paste, which was more obvious in 5%. Since the yield stress is the transition point of the substance behaves from a solid to fluid, and the plastic viscosity represents the resistance of a substance to flow [60,61], the significant reduction on yield stress and plastic viscosity further proved the improved performance of cement paste with Mmt under the separate addition method of PCE and PR. Moreover, it has been summarised that, in cementitious paste, that the addition of chemical admixture, for example, superplasticiser, can reduce both yield stress and plastic viscosity, which shows a similar trend with the addition of water [62]. The similar trend by DA and PA further indicated the improved dispersion ability of PCEs with Mmt.

### 3.3. Effect on Setting Time

**Figure 4:**
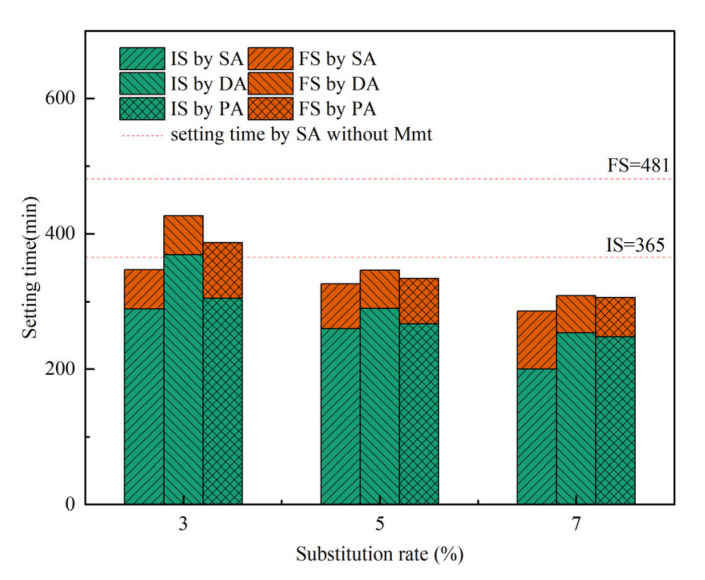
Initial setting (IS) and final setting (FS) time of cement pastes with PCE and PR under different substitution rate of Na-Mmt.

The setting time of the three addition methods with different substitution rates of montmorillonite clays are shown in Figure 4. As shown in the figure, compared to that without Mmt, the substitution of Mmt significantly decreased both initial setting (IS) and final setting (FS) of the cement paste, which was further decreased when the substitution rate increased. It can be seen from the diagram that the setting time decreases with the increase of montmorillonite substitution rate, which was closely related to the current research due to the accelerated hydration rate and condensed structure formation [63]. However, it should be noted that, at all substitution rates, the SA showed the shortest IS and FS time, while the DA exhibited the longest IS and FS time, indicating that the separate addition could not only improve the dispersion ability of cement paste but also delay the setting of the paste. Since the addition of PCE can delay the setting time of cement paste with its full function [64], compared to SA, the further delayed setting time by DA and PA further indicated that the dysfunction of PCE due to the Mmt was partially resolved.

### 3.4. Effect on Sorption of PCEs

**Figure 5:**
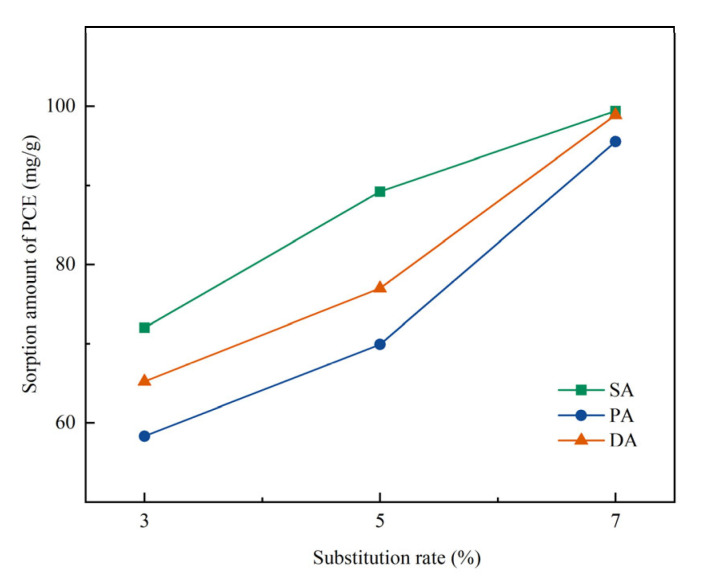
Effect of different separate addition methods on the sorption of PCE in cement with Na-Mmt.

The sorption amount of the PCEs on PR-incorporated cement with Mmt under different substitution are shown in Figure 5. As shown in the figure, regardless of the addition method, the increase of substitution ratio significantly increased the sorption of the PCE, which could be due to the large surface area of Mmt adsorbing more PCEs and the interlayer space within Mmt trapping the side chain the PCEs [22]. However, it is surprising that the sorption amount of PCE decreased in the separate addition method—it should have increased when the workability increased in the cement system [51]. This abnormal behaviour of PCE could be attributed to the intercalation of PCEs within the interlayer of Mmt, which increased the sorption of PCEs [65]. However, when separate addition was applied, the PR worked as the sacrificial agent [66], which reduced the amount of PCEs due to the intercalation. Since 70% of the sorption of PCE was attributed to the intercalation [31], which could not contribute to the dispersion of cement particles, the increased workability with a decreased sorption amount of PCE by DA and PA could occur.

### 3.5. Effect on Thickness of Adsorption Layer

**Figure 6:**
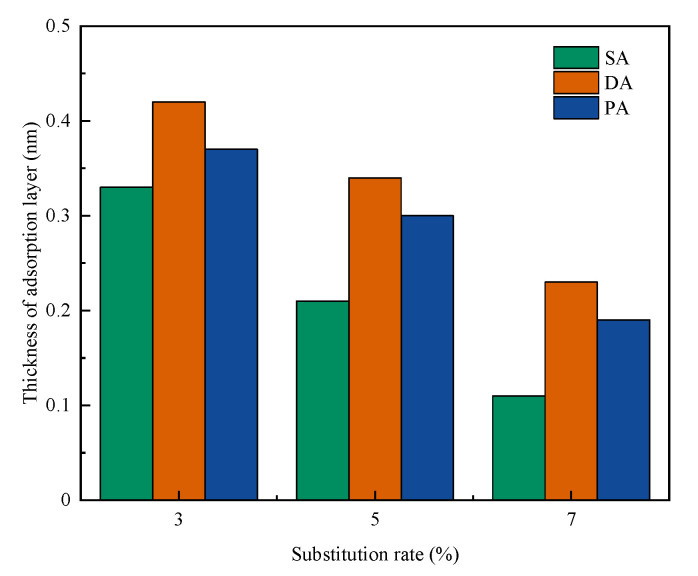
Effect of different separate addition methods on the thickness of adsorption layer of PCE in cement with Na-Mmt.

The thickness of adsorption layers of the PCEs on PR-incorporated cement with Mmt under different substitutions were conducted, and the results are shown in Figure 6. As shown in the figure, regardless of the addition method, the higher substitution ratio led to the thinner thickness of adsorption of the PCE. This result was similar to Li’s results—that the thickness of adsorption layer increased with increasing Mmt content [67], which could be due to trapping the side chain of the PCEs within the interlayer of Mmt, leading to the suppressed steric repulsion of PCE [52]. However, it is not surprising that when the separate addition method is applied, the thickness of the adsorption layer is further increased, with thickest layer being observed by DA. This could be because, prior to adding PR, the PR could firstly react as the sacrificial agent, which hinders the expansion from Na-Mmt and may enter the space of interlayer of Mmt. Therefore, the side chain of PCEs could not be trapped. Since the higher adsorption layer may lead to a better workability, the increased adsorption layer thickness by a separate addition may be applied to explain the increased workability of cement paste with Mmt.

### 3.6. Effect of Intercalation Behaviour of PCE

**Figure 7:**
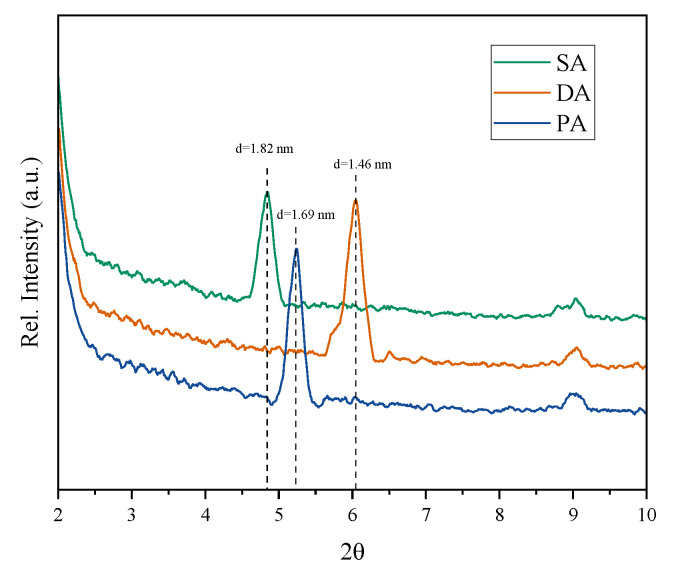
XRD pattern of Na-Mmt under different addition methods.

The intercalation between the PCE and the Na-Mmt was calculated from the d-spacing of the silicate layers (d_001_) and of the Mmt, and the XRD patterns of Na-Mmt under different addition method are plotted in Figure 7. As shown in the figure, under the SA, the d-spacing of the Na-Mmt was 1.82 nm, which was similar to the other reports of Mmt with the trapped side chain of PCEs [52]. However, when the separate addition was applied, the d-spacing was decreased to 1.69 nm by PA and 1.46 nm by DA, respectively. The reduced d-spacing indicated that the interaction due to the PCE was eliminated. Therefore, it can be deducted that the separate addition of PCE and PR can reduce the intercalation of the PCEs within the Na-Mmt, which therefore can be explained the results of higher workability (Figure 2) with higher adsorption (Figure 5) by the DA and PA. Compared with the separate addition, lower d-spacing was observed by DA than that by PA, which could be attributed to the time interval for adding PCE and PR enhancing the sacrificial behaviour of PR.

## 4. Conclusions

Based on the results, the following conclusion can be drawn as:

(1) Although the substitution of Na-Mmt showed an adverse effect on the fresh properties, the increase in the minislump and the minislump retention of the cement paste with Na-Mmt occurred when PCE and PR were separately added. Comparing different adding methods, the minislump of DA and PA is higher than of SA, with DA being better than PA.
(2) The order of PCE and the addition of retarders changed the rheological behaviour of Na-Mmt-incorporated cement pastes. The separate addition of PCE and PR reduced the thixotropic area of the cement paste with Mmt. Both the yield stress and the plastic viscosity of the cementitious materials containing Na-Mmt were reduced by adding the PCE and PR.
(3) The sorption of PCEs on cement particles with Na-Mmt was decreased by separately adding PCEs and PR, while the thickness of the adsorption layer of PCE was increased under a separate addition method.
(4) In the presence of the PR, reduced intercalation occurred for PCE with the interlayers of Mmt under a separate addition.
(5) Although the synergistic effect of PCE and PR was observed and the potential mechanism was explored, a detailed explanation on the improved performance of PCE under different addition methods was not illustrated in-depth. This needed to conduct further research.

## Figures and Tables

**Table 1:** Chemical composition (wt.%) of the cement and Mmt.

	CaO	SiO_2_	Al_2_O_3_	MgO	Na_2_O	SO_3_	Fe_2_O_3_	K_2_O	LOI
PC	64.35	21.79	4.45	2.38	N/A	2.45	3.55	0.38	1.50
Mmt	2.17	72.35	13.92	2.13	5.22	N/A	1.17	0.41	0.51

**Table 2:** Mix proportion.

Mix	Cement	Na-Mmt	Water	PCE	PR	Admixture Addition Method
1	1	0	0.29	0.6	0.05	SA
2	1	0	0.29	0.6	-	
3	1	0	0.29	-	0.05	
4	0.97	0.03	0.29	0.6	0.05	SA, DA, PA
5	0.97	0.03	0.29	0.6	-	
6	0.97	0.03	0.29	-	0.05	
7	0.95	0.05	0.29	0.6	0.05	SA, DA, PA
8	0.95	0.05	0.29	0.6	-	
9	0.95	0.05	0.29	-	0.05	
10	0.93	0.07	0.29	0.6	0.05	SA, DA, PA
11	0.93	0.07	0.29	0.6	-	
12	0.93	0.07	0.29	-	0.05	

## Data Availability

All the data associated with this study are available from the corresponding author upon request.
